# Choices for Induction of Pluripotency: Recent Developments in Human Induced Pluripotent Stem Cell Reprogramming Strategies

**DOI:** 10.1007/s12015-015-9622-8

**Published:** 2015-10-01

**Authors:** Marinka Brouwer, Huiqing Zhou, Nael Nadif Kasri

**Affiliations:** Department of Cognitive Neuroscience, Radboudumc, Nijmegen, 6500 HB The Netherlands; Department of Human Genetics, Radboudumc, Nijmegen, 6500 HB The Netherlands; Donders Institute for Brain, Cognition, and Behaviour , Centre for Neuroscience, Nijmegen, 6525 AJ The Netherlands; Department of Molecular Developmental Biology, Faculty of Science, Radboud University, Nijmegen, 6500 HB The Netherlands

**Keywords:** Human induced pluripotent stem cells, Reprogramming

## Abstract

The ability to generate human induced pluripotent stem cells (iPSCs) from somatic cells provides tremendous promises for regenerative medicine and its use has widely increased over recent years. However, reprogramming efficiencies remain low and chromosomal instability and tumorigenic potential are concerns in the use of iPSCs, especially in clinical settings. Therefore, reprogramming methods have been under development to generate safer iPSCs with higher efficiency and better quality. Developments have mainly focused on the somatic cell source, the cocktail of reprogramming factors, the delivery method used to introduce reprogramming factors and culture conditions to maintain the generated iPSCs. This review discusses the developments on these topics and briefly discusses pros and cons of iPSCs in comparison with human embryonic stem cells generated from somatic cell nuclear transfer.

## Introduction

Human embryonic stem cell (hESC) research has provided valuable information on human development by the ability to differentiate pluripotent hESCs into any human specific cell type [1–3]. This ability is especially advantageous to acquire human cells that are difficult to obtain (e.g., brain or cardiac tissue). However, research using hESCs has been limited due to strict ethical legislations [4–6]. In the last decade, several reprogramming techniques that generate human pluripotent stem cells from differentiated somatic cells were developed successfully [7–10]. These techniques circumvent the ethical legislations on hESCs.

The first reports of reprogramming somatic cells to pluripotent stem cells were from Yamanaka and colleagues, in which they showed that introducing a set of defined reprogramming factors (e.g., Oct4, Klf4, Sox2 and c-Myc, (OSKM factors)) into the somatic cells was sufficient to generate induced pluripotent stem cells (iPSCs) [7, 11]. Since then, iPSC research has attracted a lot of attention and has grown rapidly. The iPSCs provide promises in basic research and regenerative medicine, and can be used in a wide range of applications including cell-based therapies, drug screening and disease modelling. However, induced reprogramming strategies of initial studies were inefficient (~0,01–0,02 %) [7, 8, 12] and the overexpression of oncogenes such as c-Myc and Klf4 raises safety issues. Furthermore, the virus based delivery methods result in genomic integration and expression of transgenes, thereby limiting its application for clinical purpose due to risk of insertional mutagenesis. In addition, although human iPSCs share many similar features to human ESCs, epigenetic characteristics are distinct in iPSCs. Therefore, numerous protocols have been developed to improve the induced reprogramming technique [13, 14]. The variables in these protocols include the choice of the somatic cell source, reprogramming factors, delivery method and culturing conditions. Furthermore, somatic cell nuclear transfer has recently been successfully performed to generate human ESCs (NT-ESC) and provides an alternative method to confer human somatic cells to pluripotency.

**Fig. 1:**
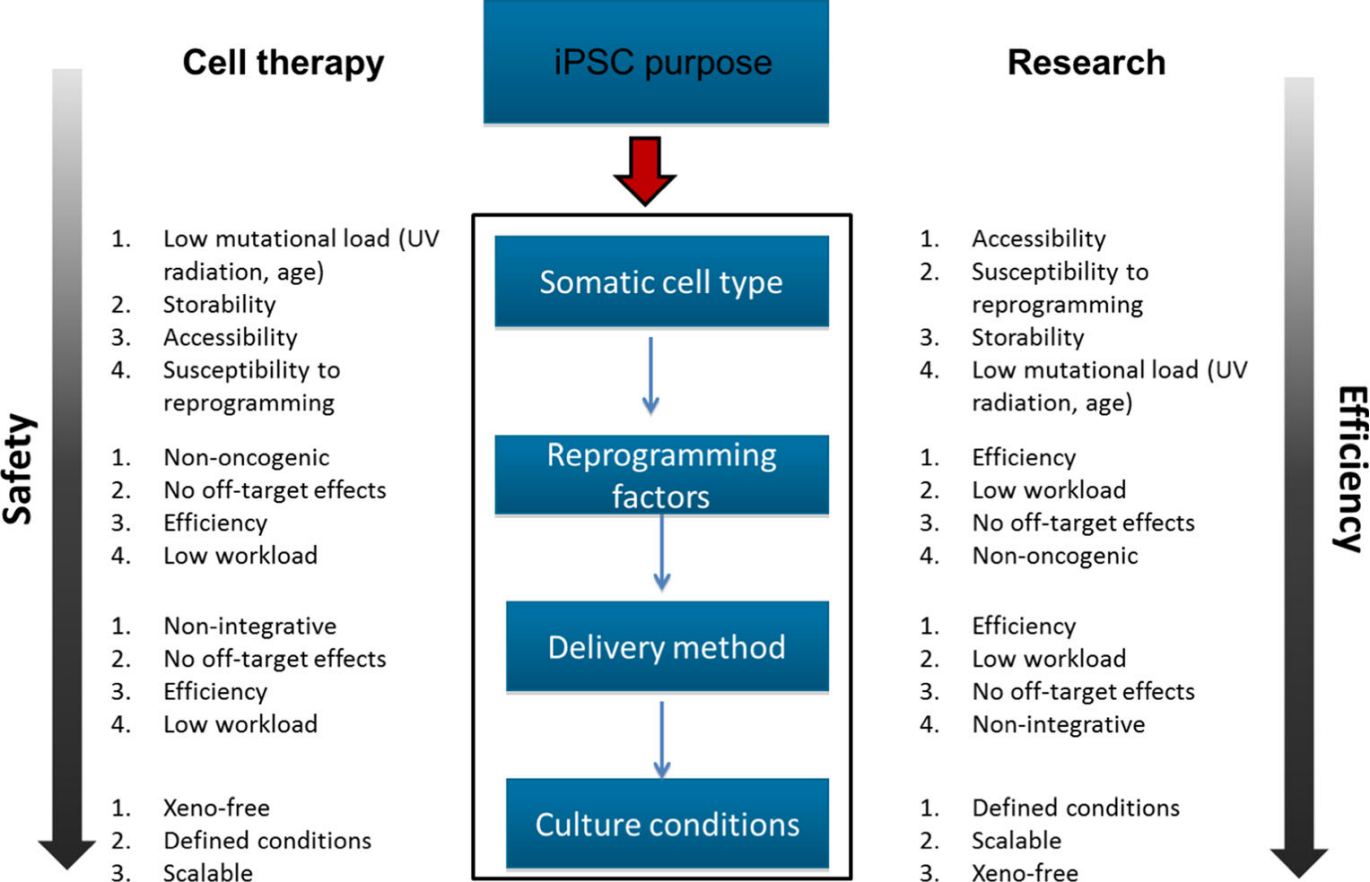
Overview of parameters influencing the reprogramming process. Depending on the purpose of the iPSCs (cell therapy or research), choices concerning the somatic cell type, reprogramming factors, delivery method and culturing conditions have to be made. With each of these aspects, we suggest to make choices on the indicated topics, depending on their ranked priority for the given iPSC purpose. Overall, when using iPSCs for cell therapy, safety should be the primary concern when making choices for the different reprogramming methods. When using iPSCs for research purposes, we recommend to choose methods which optimize the efficiency of the reprogramming process

In this review, the recent developments in strategies for the generation of iPSCs will be discussed (Fig. 1). The review will first briefly discuss the characterization of human iPSCs, and subsequently focus on the variables that influence iPSC quality and reprogramming efficiencies including cell source, reprogramming factors, delivery methods and culturing conditions. Furthermore, the comparison of generating human iPSCs and human NT-ESCs will briefly be discussed. Given the topic of this review concerning the use of human materials for research and therapy, studies using human derived iPSCs will be the focus unless stated otherwise.

## Characterization of iPSCs

**Fig. 2:**
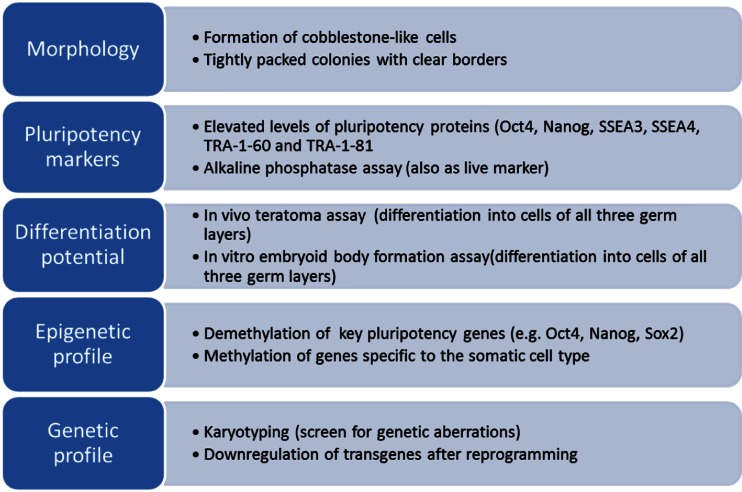
Overview of factors which should be assessed to characterize reprogrammed iPSCs. iPSCs can be characterized on five different aspects: morphology, pluripotency markers, differentiation potential, epigenetic profile and genetic profile. For each aspect factors are indicated which are important to assess the different aspects

As iPSC reprogramming efficiencies are low and the quality of the generated iPSCs is influenced by several factors, it is important to carefully characterize the iPSCs after reprogramming. Different methods have been used to characterize iPSCs (Fig. 2). The characteristic morphology of iPSCs is often used as a first indication of iPSC formation. iPSCs can be observed as small cells with large nucleus/cytoplasm ratios that form compact colonies which are defined by clear borders. In addition to cell morphology, many cellular and molecular methods are used. One of these methods includes the assessment of the presence of pluripotency marker proteins (e.g., Oct4, Nanog, SSEA3, SSEA4, TRA-1-60 and TRA-1-81), which are expressed in pluripotent stem cells [15]. Since these markers are not necessarily specific to pluripotent stem cells, the expression of multiple of the markers should be assessed in combination to determine the presence of pluripotent stem cells. Alkaline phosphatase assays can also be used to mark iPSCs. This method uses the high enzymatic activity of phosphatases in pluripotent stem cells to generate a fluorescent signal and can be used as a live marker for iPSCs [16]. In addition to these methods using morphological characteristics and cell specific markers, functional evaluation of the generated iPSCs can be performed by assessing the differentiation potential of the iPSCs. iPSCs should be able to terminally differentiate into cells of all three germ layers which can be evaluated through in vivo teratoma formation assays or in vitro differentiation through embryoid body (EB) formation into cells of the three germ layers. Furthermore, since reprogramming influences the genetic and epigenetic make-up of the cells, iPSCs should be carefully characterized for genetic aberrations and epigenetic analyses such as gene expression and DNA methylation profiles. Karyotyping is commonly used to evaluate genetic abnormalities in iPSCs. However, if transgenes are used for reprogramming, it is also important to evaluate if the expression levels of the transgenes are properly down regulated once the iPSCs are formed. For evaluation of the epigenetic profile of the iPSCs, DNA methylation patterns can be assessed. Since DNA methylation contributes to silencing of genes, it is important that the generated iPSCs show DNA demethylation at key pluripotency genes (e.g., Oct4, Nanog, Sox2), while genes specific to the donor cell type become methylated and silenced. Finally, it is important to note that the methods used to characterize iPSCs mentioned above should be used in combination rather than alone.

## Cell Source

When considering the cell source for reprogramming, somatic cells should preferentially be easily accessible, susceptible for reprogramming and the reprogramming process should ideally be highly efficient. Many human somatic cell types have been successfully reprogrammed. However, reprogramming efficiencies and kinetics vary between somatic cell types. Keratinocytes for example showed a 100 times higher reprogramming efficiency (~0,8 %) and were reprogrammed two times faster than skin fibroblasts under the same conditions [12]. Furthermore, in mice it has been shown that immature cells are more readily reprogrammed than terminally differentiated cells [17]. The comparison of reprogramming efficiencies and kinetics of somatic cell types between different studies is however complex since many of these studies are different in their choice of nuclear factors, delivery method and culturing conditions. Given the amount of human somatic cells types that have been reprogrammed using different techniques leading to different reprogramming efficiencies, an in-depth comparison on this topic is beyond the scope of this review.

Apart from the different reprogramming efficiencies and kinetics, the choice of the reprogramming factors seems also to be dependent on the somatic cell types. The OSKM factors (Oct4, Sox2, Klf4 and c-Myc) were the first set of reprogramming factors that were found to be able to reprogram somatic cells into iPSCs. However, with the exploitation of other somatic cell sources and the development of reprogramming strategies, other sets of reprogramming factors were found to be capable of reprogramming somatic cells. For example, skin fibroblasts can be reprogrammed without c-Myc. This decreases the risk of tumorigenesis, which is beneficial for therapeutic purposes, but exclusion of c-Myc also decreases reprogramming efficiency (~0,0004 %) [18]. Furthermore, some somatic cell types already endogenously express reprogramming factors necessary for reprogramming at sufficiently high levels. Melanocytes for example express Sox2 endogenously at high levels and ectopic Sox2 is therefore dispensable for reprogramming [19]. Even more, neural stem cells only need the introduction of one additional factor (Oct4) for successful reprogramming [20].

Other factors which may influence the choice of the somatic cell type are the ability to store the cells for longer periods of time and the accessibility of the somatic cell types. Since obtaining human fibroblasts is an invasive procedure, search for other cell sources more easily accessible has been performed. Cells from urine samples and (cord) blood samples for example are more easily obtained and have been successfully reprogrammed [21–25]. Cells from cord blood samples have the advantage that they may contain less somatic mutations compared to adult cells. Furthermore, (cord) blood cells have recently been shown to be reprogrammable after cryopreservation [26]. This provides opportunities for therapeutic use and personalized medicine since (cord) blood samples of individuals can be stored in blood banks and used to reprogram to hIPSC when necessary.

The choice of the somatic cell type also influences the quality of the acquired iPSCs. Given the pluripotent state of the reprogrammed cells, iPSCs derived from different somatic cell types should all be capable of differentiating into cell types of all three germ layers. However, iPSCs are known to retain an epigenetic memory of the donor cell [27–31]. Most studied epigenetic memories refer to DNA methylation and gene expression pattern. Ohi et al. showed that silencing by DNA methylation was inefficient for several genes (e.g., COMT, C9orf64 and TRIM4), which were expressed in the donor cell types, but not in human ES cells [30]. In both mouse and human models, iPSCs derived from different cell types have distinguishable gene expression patterns, DNA methylation signatures and differentiation potential [32, 33]. As a result of the epigenetic memory, iPSCs derived from donor cells of different germ layers show a differentiation preference towards cell types of the original germ layer [27, 28]. For example, iPSCs derived from blood cells more readily differentiate to hematopoietic cells, while fibroblast-derived iPSCs form more colonies when differentiating in the osteogenic direction [34]. In another mouse iPSC study, Hiler et al. developed a quantitative method to score the ability of iPSCs to form 3-dimensional retinae, and reported that iPSCs derived from rod photoreceptor cells produced more differentiated retinae than ESCs and fibroblast-derived iPSCs [35]. However the epigenetic memory seems to be a rather transient phenomenon. Continuous passaging of the iPSCs attenuates the differences between iPSCs and ESCs, in both epigenetic signature, as well as differentiation potential [32, 33]. This suggests that iPSCs lose the characteristics inherited from the parent cells over time.

So far, most human iPSCs have been derived from cell types of mesodermal origin including fibroblasts [7, 36] and other mesenchymal derived cells [37–39], several cell types from the hematopoietic lineage [23–25], amniotic fluid cells [40], adipose stem cells [41], dental pulp cells [42, 43] and urinary cells [22]. (for a more detailed overview of human somatic cell types reprogrammed so far, the reader is referred to the following database intranet.cmrb.eu/reprogramming [14]). For cells of ectodermal and endodermal origin only few cell types have been reprogrammed including keratinocytes [12, 44], neural progenitors [20, 45] and melanocytes [19] for ectoderm and hepatocytes [46] and pancreatic islet beta cells [28] for endoderm. Given the epigenetic memory of the donor cell type it may therefore be important to choose a donor cell type with the same germ layer origin as the cell type to which the iPSC’s will be differentiated.

## Reprogramming Factors

Generating iPSCs requires the introduction of pluripotency related factors into the somatic cell. Apart from the four well-known transcription factors, Sox2, Klf4, Oct4, c-Myc and the alternative combination described by the Thomson group containing Sox2, Oct4, Lin28 and Nanog [8], factors such as other transcription factors, small molecules, microRNA’s (miR) and culturing conditions have been found to increase reprogramming efficiency and iPSC quality (Table 1). Most factors have been found to target main cell signalling pathways including the TGFβ, PI3K, β-catenin, cAMP and the MAPK/ERK pathways as well as apoptosis/cell cycle related pathways. Furthermore, several factors that are known to be involved in chromatin remodelling pathways or in the hypoxia response pathway have also been reported to influence reprogramming. In this section we will discuss the influence of the different reprogramming factors on the reprogramming process.

**Table 1:** Reprogramming factors capable of reprogramming human cells

Reprogramming factors	Function	Affected pathway	Effect on pluripotency	References
Oct4	maintenance of pluripotency and self-renewal	core transcriptional circuitry	+	[7, 47]
Sox2	maintenance of pluripotency and self-renewal	core transcriptional circuitry	+	[7]
Klf4	maintenance of pluripotency and self-renewal	core transcriptional circuitry	+	[48, 49]
c-Myc	maintenance of pluripotency and self-renewal	core transcriptional circuitry	+	[7]
Lin28	maintenance of pluripotency, translational enhancer, inhibits let7	core transcriptional circuitry	+	[8]
Nanog	maintenance of pluripotency and self-renewal	core transcriptional circuitry	+	[8]
Sall4	maintenance of pluripotency and self-renewal	core transcriptional circuitry	+	[50, 51]
Utf1	maintenance of pluripotency	core transcriptional circuitry	+	[52]
p53	induces senescence, tumor suppressor	apoptosis/cell cycle	−	[52–54]
p21	induces senescence, tumor suppressor	apoptosis/cell cycle	−	[53]
P16^Ink4a^	induces senescence, tumor suppressor	apoptosis/cell cycle	−	[53, 55]
GLIS1	activates multiple pro-pluripotency pathways	core transcriptional circuitry; Wnt/β-catenin; PI3k; TGFβ	+	[56]
L-Myc	suppresses differentiation associated genes	core transcriptional circuitry	+	[57]
TGFβ	Facilitates EMT	TGFβ	+	[58]
MDM2	p53 inhibitor	apoptosis/cell cycle	+	[59]
REM2	p53 inhibitor	apoptosis/cell cycle	+	[60]
Cyclin D1	Stimulates E2F/ G1-S cell cycle transition	apoptosis/cell cycle	+	[60]
SV40 large T antigen	inhibits p53 tumor suppression	apoptosis/cell cycle	+	[54, 61]
DOT1L	histone H3K79 methyltransferase	Chromatin remodeling	−	[62]
Cx43	Promotes MET transition	E-cadherin/β-catenin	+	[63]
MBD3	histone deacetylation, chromatin remodeling	Chromatin remodeling	−	[64]
Sirt6	chromatin remodeling/ telomere maintenance	Chromatin remodeling	+	[65]
TCL1a	stimulates akt pathway	PI3k	+	[66]
RARy	Binds RAREoct, promotes Oct4 expression	core transcriptional circuitry	+	[67]
SNAIL	Promotes EMT transition	core transcriptional circuitry/TGFβ	+	[68]
Lrh-1	Binds RAREoct, promotes Oct4 expression	core transcriptional circuitry	+	[67]
RCOR2	Facilitates histone demethylation	Chromatin remodeling	+	[69]
Non-coding RNA				
miR367	inhibits EMT	TGFβ	+	[70]
LincRNA-ROR	regulates expression of core transcriptional factors	core transcriptional circuitry	+	[71, 72]
miR302	inhibits EMT/stimulates oct4 expression	TGFβ; core transcriptional circuitry; apoptosis	+	[70, 73, 74]
miR766	Inhibits Sirt6	Chromatin remodeling	−	[65]
miR200c	inhibits EMT/TGFβ pathway	TGFβ	+	[75]
miR369	inhibits EMT/TGFβ pathway	TGFβ	+	[75]
miR372	inhibits EMT/TGFβ pathway	TGFβ	+	[76]
Let7	regulates expression of core transcriptional factors and prodifferentiation genes	core transcriptional circuitry/TGFβ	−	[77, 78]
miR19a/b	inhibits PTEN	PI3k	+	[79]
Small molecules				
Vitamin C	alleviates cell senescence/antioxidant	Hypoxia response	+	[80]
Valproic acid	inhibits histone deacetylases	Chromatin remodeling	+	[81]
CHIR99021	GSK3-inhibitor	PI3k; Wnt/β-catenin	+	[82]
Parnate	lysine-specific demethylase 1 inhibitor	Chromatin remodeling	+	[82]
SB431542	ALK5/TGFβ receptor inhibitor	TGFβ	+	[83]
PD0325901	MEK inhibitor	MAPK/ERK	+	[83]
BIX-01294	Methyltransferase G9a inhibitor	Chromatin remodeling	+	[45]
Lithium	GSK3-inhibitor	PI3k; Wnt/β-catenin	+	[84]
Maxadilan	downregulates Caspase3 and 9, anti-apoptotic	apoptosis	+	[85]
8-Br-cAMP	Protein kinase A activator	cAMP	+	[86]
A-83-01	ALK5/TGFβ receptor inhibitor	TGFβ	+	[87]
Tiazovivin	promotes survival, ROCK inhibitor	PI3k	+	[83]
Y-27632	promotes survival, ROCK inhibitor	PI3k	+	[88]
EPZ004777	DOT1L inhibitor	Chromatin remodeling	+	[62]
DAPT	Inhibits Notch/ increases core transcription factor expression/ inhibits p53 pathway	core transcriptional circuitry/ apoptosis	+	[89]

Reprogramming factors include non-coding RNA’s and small molecules. Each factor has a specific function in one or more pathways and has to be upregulated (+) or downregulated (−) to induce reprogramming

The transcription factors that have been used so far to reprogram human somatic cells have been found to play important roles in maintenance of pluripotency and self-renewal by acting through complex transcriptional networks [57, 90]. Nearly all of the transcription factors that have been used to reprogram human somatic cells are part of a core pluripotency circuitry. Within this circuitry, two distinct modules have been suggested to regulate transcription [90]. One involves the Oct4-module, which also includes Sox2, Nanog, Sall4 and Utf1 while the other involves the cMyc-module. Though suggested to be distinct, crosstalk between the different modules exist. Klf4 [48] and GLIS1 [56] for example are thought to be upstream regulators of both the Oct4- and cMyc-module.

Furthermore, although not functioning as transcription factors, Lin28 and non-coding RNA’s Let7 and lincRoR have been found to be directly involved in the core transcriptional pathway and increase reprogramming efficiency (~2-fold increase compared to OSKM alone) [71, 77]. Lin28 is a RNA binding protein and has been found to mediate translation of Oct4 [91] as well as the inhibition of miR Let7, which is known to promote expression of pro-differentiation genes [77]. Additionally, lincRoR is a long non-coding RNA that has been found to regulate expression of core transcriptional factors [71, 72]. For a more detailed overview of the core transcriptional network in human pluripotent stem cells the reader is referred to the following database: www.StemSight.org [92].

**Fig. 3:**
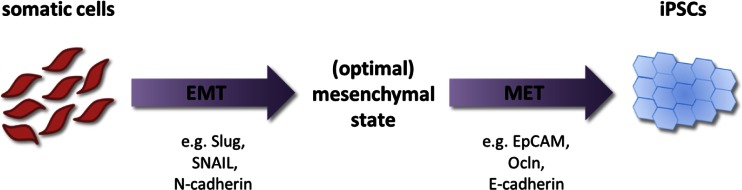
Sequential introduction of the OSKM factors induces EMT-MET dependent reprogramming. Upon sequential introduction of OSKM (in the order OK, M, S), somatic cells undergo an initial EMT phase where mesenchymal genes including Slug, SNAIL and N-Cadherin are upregulated. Once the cells reach an optimal mesenchymal state after EMT they undergo MET by downregulating the mesenchymal genes and upregulating epithelial genes including EpCAM, Ocln an E-Cadherin

For the clinical application of iPSCs, it is desired to have the reprogramming efficiency as high as possible. So far reprogramming efficiencies with OSKM transcription factors are rather low which is probably not yet optimal for clinical use. Addition to or replacement of the OSKM factors with the previously discussed factors involved in the core transcriptional pathway have shown to reach similar or increased reprogramming efficiency compared to when using the OSKM factors alone. Recently, it was shown that sequential introduction of the OSKM factors increased reprogramming efficiency ~5-fold compared to simultaneous introduction [58]. Liu et al. showed that reprogramming is a dynamic process where the OSKM factors influence both the epithelial to mesenchymal transition (EMT) and the mesenchymal to epithelial transition (MET) [58]. EMT and MET play important roles in embryonic development and cancer progression and involve up- or downregulation of genes specific to epithelial or mesenchymal cells [93, 94]. Pluripotent stem cells more closely resemble epithelial cells showing tight compact colonies and the cells express the epithelial marker E-cadherin. However, the fibroblasts which are widely applied for reprogramming are of mesenchymal origin and express mesenchymal markers including N-cadherin and Slug [58, 95]. This indicates that the fibroblasts may have to undergo at least a MET phase to reprogram into iPSCs. The role of MET in iPSC reprogramming is further supported by the ~3-fold increase in reprogramming efficiency after the addition of Connexin43 to the reprogramming cocktail compared to reprogramming with OSKM alone [63]. Connexin43 is thought to mediate MET by regulation of the expression of E-cadherin, a marker of MET. Interestingly, addition of the EMT promoting factor TGFβ to the reprogramming cocktail throughout the reprogramming process decreased reprogramming efficiency, but addition of TGFβ during the first 2 days of reprogramming increased the efficiency ~2-fold [58]. Similarly, overexpression of the EMT promoting factor SNAIL at early reprogramming stage increased reprogramming efficiencies. Furthermore, Unternaehrer et al. showed that overexpression of SNAIL enhanced reprogramming of mesenchymal fibroblasts as well as epithelial keratinocytes, indicating that somatic cells of both mesenchymal and epithelial origin may need to undergo an EMT phase for reprogramming [68]. Therefore, these findings indicate that reprogramming may consist of at least two phases, an initial EMT phase followed by a MET phase (Fig. 3). Liu et al. suggest that the mesenchymal fibroblasts can reach a more optimal mesenchymal state during the EMT phase, making the cells more susceptible for the following MET phase, thereby increasing the reprogramming efficiency. Further investigation of this dynamic EMT-MET process and the role of different factors herein may therefore be used to optimise the reprogramming mechanisms, thereby increasing reprogramming efficiency.

Another set of factors that have been used to reprogram human somatic cell types are involved in the apoptosis/cell cycle pathway. Several of these proteins including p53 are tumour suppressors; they inhibit growth and promote senescence, functions that are undesirable for the reprogramming process. Targeting these type of proteins with shRNA’s during reprogramming have shown to increase reprogramming efficiency (between ~2- and 10-fold increase compared to OSKM alone) [52, 53, 55]. However, given the risk of off-targets effects, the use of shRNA’s may not be a useful technique in a clinical setting. Therefore, other ways of inhibiting the apoptosis pathway have been investigated. Overexpression of proteins that are known to inhibit p53 (such as MDM2, SV40 large T antigen and REM2) have been shown to increase reprogramming efficiency as well (between ~1.5- and 23-fold increase compared to OSKM alone) [54, 59–61].

Chromatin remodelling is an important step in the reprogramming process. As discussed before, DNA methylation is important to silence genes specific to the somatic cell type and incomplete silencing of these genes causes an epigenetic memory of the donor cell in the reprogrammed iPSCs. DOT1L [62], MBD3 [64], RCOR2 [69], Sirt6 and Sirt6-inhibitor miR766 [65] are involved in chromatin remodelling and have been shown to affect reprogramming efficiency when overexpressed or inhibited. DOT1L is a histone H3K79 methyltransferase, which activates genes upon methylation. Although seemingly contradicting, inhibition of DOT1L increased reprogramming efficiency ~3-fold compared to OSKM alone [62]. The authors suggest that DOT1L inhibition during the initial phase of reprogramming facilitates loss of H3K79Me2 on genes associated with the donor cell type thereby stimulating repression of these genes and promoting reprogramming. Sirt6 on the other hand is known to deacetylate H3K9Ac and H3K56Ac thereby repressing gene expression. Overexpression of Sirt6 resulted in increased reprogramming efficiency (~2-fold compared to OSKM alone), likely by facilitating repression of donor cell type specific genes [65]. Likewise, RNA interference of Sirt6-inhibitor miR766 increased reprogramming efficiency (~1.5-fold compared to OSKM alone) [65].

Although overexpression or inhibition of other factors in addition to (a subset of) the OSKM factors can increase reprogramming, the overall efficiency remains low and is considered a stochastic process. Rais et al. argued that inhibition of MBD3, a member of the MBD3/NuRD complex that represses gene activity by deacetylation, reprograms donor cells types into iPSCs in a deterministic fashion rather than stochastic [64]. The OSKM factors themselves are thought to recruit the MBD3/NuRD complex to the downstream target genes of the OSKM factors thereby inhibiting their activity. Rais et al. found that inhibition of MBD3 in addition to overexpression of the OSKM factors yielded a reprogramming efficiency of nearly 100 %. However, a recent report showed contradicting evidence that MBD3/NuRD complex is required for the reprogramming of mouse somatic cells [96]. Although chromatin remodelers can increase the reprogramming efficiency, their use in reprogramming should be considered with care, since they do not target specific genes, but rather affect the entire genome. They may therefore cause yet unknown and perhaps undesired side-effects due to off-target chromatin remodelling.

As mentioned before, non-coding RNA’s can be used to increase reprogramming efficiency. Most of the microRNA’s used to increase reprogramming efficiency inhibit the TGFβ pathway, thereby inhibiting EMT [70, 73, 75, 76]. miR302 alone or combinations of microRNA’s (miR302/miR367 [70] and miR302/miR200c/miR369 [75]) have been shown to be very potent in reprogramming as they can fully replace the original OSKM transcription factors, obtain a similar [75] or higher [70] reprogramming efficiency (~2-fold increase) compared to OSKM and do not require addition of other factors for reprogramming. All combinations involve miR302, which has been shown to stimulate the expression of Oct4/ Sox2 and Nanog as well as inhibiting several factors that stimulate DNA methylation [73] and stimulating tumour suppressor related pathways [74]. MicroRNA’s have the advantage of specifically targeting multiple pathways and as seen for miR302 may therefore reduce the amount of factors to be introduced to induce pluripotency.

Finally, the use of small molecules during reprogramming has also been shown to significantly improve reprogramming efficiency and iPSC quality. The small molecules that have been used to improve reprogramming of human somatic cells target several different signalling pathways and affect chromatin remodelling, which is extensively reviewed by Federation et al. [97]. Unlike the other factors described previously, small molecules do not require any additional delivery methods to introduce them into the cells. This makes the reprogramming process less labour intensive and enables strict control of exposure of cells to the factors. However, although the small molecules have a relatively high specificity for their targets, non-specific effect may cause cellular toxicity. Development of highly specific small molecules may therefore decrease this toxicity and further improve reprogramming efficiency. Recently, a cocktail of small molecules alone have shown to be able to reprogram mouse embryonic fibroblasts with a comparable efficiency as when using transcription factors [98]. However, it is not yet established if reprogramming using small molecules alone can also be achieved for human cells.

In summary, the choice of the reprogramming factors is dependent on many factors. First, the choice of the somatic cell type may affect the choice of the reprogramming factors used. Some somatic cell types exhibit a sufficiently high endogenous expression of reprogramming factors that exogenous introduction is unnecessary (e.g., Sox2 expression in melanocytes [19]). Apart from this, the choice of the nuclear factors also depends on the purpose of the acquired iPSC’s. Several factors are oncogenic and may form a risk when genomic integration based delivery methods are used, which is undesired for clinical purposes but is not necessarily problematic for disease modelling, for example. In addition, the choice of nuclear factors affects the efficiency of reprogramming. As mentioned before, the reprogramming efficiency decreases when fibroblasts are reprogrammed with only Sox2, Klf4 and Oct4 when compared to the addition of the oncogene c-Myc to the reprogramming cocktail [18]. MicroRNA’s have been shown to be able to fully replace nuclear factors and may provide a more effective way of reprogramming than traditional nuclear factor reprogramming. Furthermore, small molecules can effectively increase reprogramming efficiency or replace several nuclear factors. So far, reprogramming using small molecules alone has only been established for mouse cells [98]. However if this method is also effective on human cells, it may provide a method for reprogramming iPSCs for clinical purpose.

## Delivery Method

In addition to the choice of the somatic cell type and the reprogramming factors, it is important to select proper delivery methods for the reprogramming factors to enter the cells. The choice of the delivery method is strongly dependent on the choice of the reprogramming factors. Small molecules for example have the major advantage that they do not require any additional delivery method. The other factors can be introduced into the somatic cells as DNA, RNA or proteins. DNA can be delivered into the cells using a variety of methods including viruses [7, 99, 100], transposons [101, 102], bacteriophages [103] and episomal vectors [104, 105] (Table 2). RNA can be delivered using viruses [113] or directly as RNA molecules [108] and proteins can also be directly introduced into the cells [112] (Table 2). In this section we will discuss the different delivery methods used to reprogram somatic cells and how this affects reprogramming efficiency and quality.

**Table 2:** Delivery methods used to deliver reprogramming factors into human somatic cells. Delivery methods can be divided in integrating and non-integrating methods

	Delivery method	Advantages	Disadvantages	References
Integrative	Retrovirus	Very efficient, widely applied	Genomic integration, cell type specificity, requires cell division	[7, 12]
	Lentivirus	Very efficient, does not require cell division, infects wide range of cell types, inducible/excisable	Genomic integration	[99, 106]
	Transposon	Relatively efficient, xeno-free, excisable	Genomic integration, risk of reintegration	[101, 102]
	Bacteriophage	Integrates in intergenic regions	Genomic integration	[103]
	Zinc finger nucleases	Targeted integration, excisable	Genomic integration	[107]
Non-integrative	mRNA	No genomic integration, relatively efficient	Needs multiple transfections, triggers immune response	[108–111]
	Episomal vector	No genomic integration, relatively easy	Very inefficient, requires multiple transfections, risk of genomic integration	[104]
	Protein	No genomic integration	Very inefficient, requires multiple transfections, requires high levels of proteins	[112]
	Adenovirus	No genomic integration	Very inefficient, requires multiple infections	[100]
	Sendai virus	No genomic integration, infects wide range of cell types, easily removable	Requires multiple viruses containing one factor each	[113, 114]
	Minicircle DNA	No genomic integration, relatively easy, small constructs, xeno-free	Very inefficient, requires multiple transfections	[105]

Each method has advantages and disadvantages for use in reprogramming

Retroviruses provide a relatively easy and efficient way of introducing factors into the somatic cells. However, retroviruses randomly integrate into the hosts’ genome and may therefore lead to insertional mutagenesis in the obtained iPSCs. Furthermore, the use of retroviruses for reprogramming is dependent on the choice of the somatic cell type. Retroviruses require cell division to integrate in the genome. A slow-dividing somatic cell type will therefore reduce reprogramming efficiency compared to fast-dividing cell types. Furthermore, different subtypes of retroviruses have been used for reprogramming and these subtypes do not infect all types of cells with the same efficiencies [12, 115]. It is therefore important to choose a subtype of retroviruses that is capable of efficient infection of the chosen somatic cell type. It is also important that once the iPSCs are formed, the integrated transgenes will be silenced. The transgenes delivered by retroviruses will be silenced over time, but silencing is not always efficient and some transgenes may not be silenced at all [116]. Furthermore, the transgenes that are efficiently silenced will remain in the genome and may be reactivated later on [115, 116].

Initially, several different retroviruses needed to be generated, each containing only one reprogramming factor [7]. This method leads to multiple integration sites thereby increasing the risk of insertional mutagenesis. Furthermore, the site of integration is uncontrollable and all factors need to be expressed to induce reprogramming. To overcome these problems, poly-cistronic lentiviruses were developed that contain all reprogramming factors in one vector [117]. In these viruses, the reprogramming factors are driven by a single promoter and separated by self-cleaving 2A peptide sequences. This significantly reduces the amount of integration sites in the somatic cells and provides a safer method of generating iPSC.

Unlike retroviruses, lentiviruses do not require cell division to integrate into the hosts’ genome. Furthermore, lentiviruses have the ability to infect a broader range of cell types than retroviruses. The use of a lentivirus is therefore less dependent on the choice of the somatic cell type compared to the use of retroviruses. Like retroviruses, lentiviruses integrate transgenes in the hosts’ genome, which may have the disadvantages of insertional mutagenesis, inefficient silencing or transgene reactivation as is seen with retroviruses. Additionally, the effects of inefficient silencing and transgene reactivation can be overcome with the use of excisable poly-cistronic lentiviral vectors [118, 119]. By flanking the transgene sequence with LoxP sites, transgenes can be successfully excised when exposed to Cre recombinase. However, using a CreLoxP system increases the workload of reprogramming due to additional cloning of LoxP sites and screening for proper excision. Furthermore, excision using a CreLoxP system leaves a scar in the genome, which still may result in insertional mutagenesis.

Another way of controlling lentiviral transgene expression is with the use of doxycycline inducible lentiviruses [99, 106]. In these viruses a doxycycline inducible promoter drives the transgenes. This not only allows for temporal regulation of transgene expression, but also allows for generation of ‘secondary’ iPSCs. Since the inducible system is still in the hosts’ genome once IPSCs are generated and differentiated into secondary fibroblasts, the fibroblasts can then be reprogrammed upon treatment with doxycycline into secondary iPSCs. Reprogramming secondary fibroblasts into secondary iPSCs therefore does not require reinfection with viruses. Furthermore, the population of iPSCs that are obtained are more homogeneous than virus-infected iPSCs [99, 106] and can be used to investigate the mechanisms of reprogramming [120].

To circumvent the risks associated with insertional mutagenesis integration-free human iPSCs have been generated using several methods, including adenovirus, Sendai virus, the piggyBac system, minicircle vector, episomal vectors, direct protein delivery and synthesized mRNA (Table 2). One of these methods uses replication-defective adenoviruses to deliver reprogramming factors into the cells [100]. Although this overcomes the problem of insertional mutagenesis, since the virus is not integrated in the genome it will be diluted over time due to host cell division. Reprogramming using adenoviruses therefore requires multiple viral infections throughout the reprogramming process [100]. Furthermore, the production of adenoviruses is labour-intensive and reprogramming efficiencies using adenoviruses are lower compared to lenti- or retroviruses.

Apart from viruses, reprogramming genes can be delivered into cells using several other methods. Two of these methods, transposons and bacteriophages, depend on integration of the transgenes into the genome. The PiggyBac (PB) transposon was the first transposon system to be used to generate human iPSCs [101]. In general, this method depends on a PB transposase which cuts inverted terminal repeat sequences that flank all the reprogramming transgenes separated by 2A sequences and pasts this into TTAA-sites in the hosts genome. Similarly, the PB transposon containing the transgenes can be cut out of the genome once reprogramming is established using the PB transposase. Although excision is also possible with the use of lentiviruses as discussed previously, excision of PB transposons does not leave genetic scars and therefore forms no risk for insertional mutagenesis. Furthermore, unlike using viruses, PB transposons can be used for reprogramming of any cell-type and they allow the generation of xeno-free iPSCs.

There are also several draw-backs to the use of PB transposons in reprogramming. First, there is a risk that the transposon will integrate back into the genome. Furthermore, the human genome contains endogenous PB transposon elements [121, 122], which may respond to the PB transposase that is introduced to excise the transgene transposon. Additionally, a considerable amount of transposon integration sites are found within transcription units [123]. Recently, the Sleeping Beauty (SB) transposon was used to reprogram human cells [102, 124], which can overcome several issues of the PB transposon. First, the SB transposon integrates less in transcription units than PB transposons [123]. Also, there are no SB-like elements found in the human genome and the SB transposase can therefore not affect endogenous transposable elements. Furthermore the SB100X transposase is more efficient than the PB transposase. Nevertheless, the use of transposons in general requires multiple rounds of excision, and therefore remains a risk of reintegration and overall reprogramming efficiencies are relatively low compared to the use of viruses.

So far, the described methods that depend on integration in the genome form a risk due to possibility of integration into transcriptional regions thereby disturbing endogenous gene expression. Bacteriophages use an integrase enzyme to insert their DNA into bacterial DNA by reactions of phage attachment sites (attP) with bacterial attachment sites (attB). AttB-like sites have also been described to be present in the human genome. Most of these sites have intergenic locations, although some are located in introns [103]. More recently, zinc finger nucleases were designed that could be used to generate as well as enable engineering of human iPSCs [107]. These also integrate into the genome, but in contrast to randomly integrating methods, genomic locations can be specifically targeted for integration using zinc finger nucleases. Furthermore, combining this system with the CreLoxP system allows for removal of the transgenes after reprogramming [107].

Apart from the methods described above, DNA encoding the reprogramming factors can be introduced into the cells by transient transfection of DNA molecules. Like adenoviruses, this method does not depend on integration into the hosts’ genome. Furthermore, this technique is relatively easy to use compared to the use of viruses for gene delivery. The DNA can be transfected into the cells as plasmids [104, 125–127] or as minicircle DNA [105, 128]. Minicircle DNA has the advantage over plasmids that they are small constructs that only contain the eukaryotic genetic material. Since they do not contain the bacterial backbone of the original plasmid, they may be less immunogenic than plasmids. However, reprogramming efficiencies using these minicircles are extremely low [128]. Furthermore, both plasmids and minicircle DNA generally require multiple transfections during reprogramming since their expression is only transient. Although recently a minicircle vector was developed (CoMIP), which was able to reprogram human somatic cells with only a single transfection, the use of this vector did not markedly increase reprogramming efficiencies [129]. Since transfection efficiencies are also dependent on the somatic cell type [130], this method may not be optimal in all reprogramming conditions. Although considered a non-integrating method, transgenes have been detected in the host genome upon transfection with plasmids [126].

Currently, episomal reprogramming has emerged as one of the preferred non-integrating methods. Episomal reprogramming is based on the Epstein-Barr Nuclear Antigen-1 (oriP-EBNA1) that has the ability to replicate in synchrony with the host genome by attaching to the host chromatin and replicating with each cell cycle division. The oriP/EBNA1 vector undergoes stable extrachromosomal replication only once per cell cycle, without integrating into the host genome. This results in an extended presence within a host cell without integration or modification of the host’s genome. Similar to previous discussed non-integrating methods the efficiency for hiPS generation with episomal reprogramming remains low [104, 131]. The efficiency has considerably been improved (10–100 fold) by suppressing p53 and using non-transforming L-Myc instead of c-Myc, during the reprogramming process [57]. Important advantages of episomal reprogramming are the rapid loss reprogramming agents and the high reliability of hiPSC generation from fibroblast and blood samples [132, 133]. However, the use of the p53 shRNA is problematic for therapeutic use [57].

To completely avoid DNA integration into the hosts’ genome during reprogramming, reprogramming methods introducing mRNA into cells rather than DNA have been developed. Sendai viruses have been used to successfully introduce RNA reprogramming factors into somatic cells [113, 114]. The Sendai virus efficiently introduced negative-strand single stranded RNA of reprogramming factors into the cells. Importantly, Sendai viruses can infect a wide range of somatic cell types and is therefore less dependent on somatic cell type choice compared to retroviruses. Furthermore, the viral particles can be removed by antibody-mediated negative selection against surface protein HN on the virus [113]. Point mutations in polymerase-related genes of the Sendai virus enabled controllable temperature-sensitive Sendai viruses that could be removed from the hosts by temperature increase [114]. However, these viruses only contain up to one of the reprogramming factors each. Reprogramming with four factors would therefore require four different viruses, which may cause differences in stoichiometry of factors between hiPSC clones. Recently, a new type of temperature-sensitive Sendai virus was developed (TS12KOS) which contains three reprogramming factors (Klf4, Oct4 and Sox2), thereby reducing the number of different viruses necessary for reprogramming. In combination with a temperature-sensitive Sendai virus containing c-Myc, TS12KOS was shown to effectively and more efficiently induce reprogramming compared to the combination of four different Sendai viruses containing only one reprogramming factor each [134]. An efficient Sendai virus that contains all four reprogramming factors (Klf4, Oct4, Sox2 and c-MYC) in one virus have also been developed. This Sendai virus (SeVdp) was developed from the temperature sensitive variant, but can be removed from the hosts using siRNA. The SeVdp virus, containing all four Yamanaka factors, have been shown to successfully reprogram human dermal fibroblasts of patients with Fabry disease. [135, 136]. Although SeV reprogramming is efficient, reliable and footprint-free a major drawback for the therapeutical use comes from the fact that SeV is currently not available commercially as a cGMP-grade reagent for reprogramming [131].

Apart from delivery by Sendai viruses, RNA’s can be directly delivered into somatic cells by transfection as synthetic modified mRNA. These mRNA’s can be capped with a 5′ guanine cap to increase RNA half-life and translation efficiency. However, a fraction of the synthetic RNA’s remains uncapped and bears 5′triphosphates, which can induce innate immune responses. To reduce this response, synthetic RNA’s can be treated with phosphatase prior to transfection. Furthermore, substitution of ribonucleoside bases cytidine and uridine for the modified ribonucleosides 5-methylcytidine and speudouridine respectively significantly reduced the immune response. Addition of interferon inhibitors to culturing media helps to reduce immunogenicity [108]. Although these methods have been used during reprogramming of cells on feeder cells, more recently feeder- and xeno-free iPSCs reprogrammed with modified mRNA’s have been established [109].

Although the immune response upon reprogramming with mRNA’s can be reduced, other implications limit the use of RNA’s for reprogramming. RNA’s have short half lives, reprogramming therefore requires frequent transfections during the reprogramming process to sustain reprogramming factor expression levels in the cells. Electroporation of the RNA’s is therefore not the most suitable method for transfection and other transfection methods may not work as efficiently on each cell type [109]. To reduce the transfection frequency Yoshioka et al. developed a self-replicating positive-strand RNA replicon based on the Venezualan equine encephalitis (VEE) virus RNA replicon [110]. With this method, cells require only one transfection round and the replicons are lost over passaging of the established iPSCs. Furthermore, this replicon contains all reprogramming factors thereby retaining reprogramming factor ratios. However, this method also induced an immune response, which has to be counteracted by interferon inhibitors [110].

Finally, reprogramming factors can be introduced directly as proteins into the somatic cells [112]. Like direct delivery of mRNA, direct delivery of reprogramming proteins requires multiple transfections to gain sufficiently high intracellular levels for reprogramming. Kim et al. produced the reprogramming proteins in HEK293 cells and used their extracts to treat somatic cells. Since macromolecules have implications with transmembrane transport, Kim et al. coupled a cell-penetrating peptide (CPP) to the proteins to be delivered. However, reprogramming efficiencies using cell extracts containing the reprogramming proteins is very inefficient. Possibly, purification of the proteins may increase reprogramming efficiency.

Recently Schlaeger et al. systematically compared the most widely used integration free methods such as Sendai-viral (SeV), episomal (Epi) and mRNA transfection methods using a number of criteria [131]. Although all methods resulted is high-quality hIPSC cells there are differences at the level of aneuploidy rates, reprogramming efficiency, reliability and workload. In summary, the choice of the delivery method depends on the purpose of the generated iPSCs and may also depend on the choice of the somatic cell type. Although integrating methods have generally higher reprogramming efficiencies than non-integrating methods, use of iPSCs in clinical settings will require non-integrating methods to obtain high quality iPSCs. Reprogramming factors can be introduced into cells as DNA, RNA or proteins. DNA-based methods are most efficient although even non-integrating methods (episomal vectors) may integrate into genomes to some extent. Furthermore, RNA-based methods are relatively efficient and do not integrate in the genome, but they are also highly immunogenic. Finally, protein-based methods are promising but yet extremely inefficient.

## Culture Conditions

Manipulation of the culture environment of iPSCs has shown to be able to improve reprogramming efficiencies and iPSC quality as well. FGF2 and human LIF for example are known to inhibit differentiation and enable long-term culture of human pluripotent stem cells [82, 137]. More recently, addition of CCL2 to the cultures has been shown to induce a hypoxia response in the cells and enhance expression of pluripotency genes [138]. The hypoxia response has been previously related to increased reprogramming efficiencies as well. Transient hypoxia conditions during reprogramming have been shown to increase reprogramming efficiency [42, 139]. Hypoxia during early stage of reprogramming is thought to induce several factors (HIF1a and HIF2a) that shifts oxidative metabolism of somatic cells to the glycolitic metabolism of pluripotent cells while hypoxia during later stages of reprogramming inhibits the reprogramming process [140]. However, though increasing the reprogramming efficiency, it should also be taken into consideration that hypoxia also causes cellular toxicity.

Apart from addition of growth factors, iPSCs require specific culturing conditions for growth and maintenance, as is discussed in detail by Chen et al. [141]. In general, iPSCs can be cultured in three ways: (1) colonies, (2) non-colony monolayers and (3) suspension cultures. The first two methods require a certain extracellular matrix for the cells to grow on, while in suspension cultures cells can grow either with or without a matrix. Feeder cells are the most commonly used form of extracellular matrix for the growth and maintenance of iPSCs as colonies. However, these feeder cells are usually xenobiotic (e.g., MEFs) and the composition of the compounds, which the cells excrete, is not fully defined. For clinical purpose it is important to culture the iPSCs under fully defined conditions. Therefore, the use of other feeder-free extracellular matrices have been investigated such as Matrigel [7, 8], but also both feeder- and xeno-free matrices such as laminin [142, 143], vitronectin [144] or synthetic surfaces [145].

Matrigel has been used to sustain non-colony monolayers of iPSCs. These monolayers have been shown to increase cell viability and cells can be grown on larger scale then when using the colony-based method [146, 147]. Likewise, suspension cultures enable scalable production of iPSC. As mentioned before, iPSC cultures in suspension do not require extracellular matrices [148, 149]. However, as a result, the cells are subject to shear force that may cause damage [150]. Addition of coated microcarriers as a substrate for the cells and microencapsulation may reduce this shear force, but so far this has only be used on hESC [151, 152].

Since culture media often contain xenobiotic or chemically undefined substances, fully xeno-free, defined culture media have been developed including Essential 8 (E8) medium, TeSR2 medium and NutriStem XF/FF medium [144, 153]. Each of these different media have been successfully used to culture human iPSCs on xeno-free matrices. For example, E8 medium can be used for both adherent cultures (using vitronectin as xeno-free matrix) and cell aggregate suspension cultures in spinner flasks [154]. Furthermore, adherent iPSC colonies cultured in E8 medium can be easily passaged by addition of EDTA after removing the medium [155]. EDTA can be used to passage iPSC colonies as loose aggregates similar to dispase. The advantage of EDTA is that it is a defined chemical and does not show batch-to-batch variability like enzymes such as dispase [155]. Recently, culture conditions for resetting human ESCs to the naive state have been reported [156, 157]. These studies provide opportunities for obtaining human iPSCs at the ground-state pluripotency.

Taken together, once iPSCs are obtained, specific culturing conditions are required for their maintenance and growth. Apart from the addition of growth factors to the medium, the conditions in which the iPSCs grow and the substrate they grow on are important variables. The use of iPSCs in clinical settings require cells to be cultured under fully defined xeno- and feeder-free conditions and the culture method should allow for scalable production of iPSCs.

## Comparison to Somatic Nuclear Transfer-Derived-ESCs

Other techniques have been explored to reprogram somatic cells into stem cells other than by introduction of reprogramming factors into the cells. One of these techniques is somatic cell nuclear transfer (SCNT), which is based on the transfer of the nucleus of a somatic cell into an enucleated oocyte. The cytoplasmic content of the oocyte is sufficient to reprogram the transferred nucleus to a pluripotent state, thereby generating ES-like cells. This technique was the first to be used to clone animals, but human ES-like cells have only recently been established with this technique [9, 10, 158]. Like for induced reprogramming, SCNT is an inefficient process. In a recent study on generating ESCs from SCNT (SCNT-ESCs), Ma et al. showed that DNA methylation and transcriptome patterns of SCNT-ESCs more closely resemble those of human ESCs than those of iPSCs, whereas iPSCs retained residual DNA methylation patterns that are typical of parental somatic cells [159]. A possible explanation for the apparent closer resemblance of SCNT-ESCs compared to iPSCs is the use of an oocyte to reprogram the DNA of the somatic cell. The oocyte may contain all physiological levels of factors necessary for reprogramming and therefore be more efficient than the artificial iPSC reprogramming technique. However, another recent report showed that SCNT-ESCs and iPSCs have similar gene expression and DNA methylation profiles, as well as comparable levels of genomic aberrations such as coding mutations and imprinted gene expression defects [160]. Although further investigations are necessary to resolve the differences in these studies, these findings suggest that NT-ESCs and iPSCs have similar properties and application potentials. In addition, the major disadvantage of generating SCNT-ESCs is that it is dependent on donation of oocytes from young women, and the procedure is sophisticated. Therefore, iPSCs may still be a preferred method to provide a large number of pluripotent stem cells in most laboratories, especially for disease modelling studies.

## Future Directions

Although progress has been made towards the establishment of safer and more efficient reprogramming techniques, there are still several remaining limitations for the generation of clinical grade iPSCs. One of the most important is the rate of mutagenesis during the reprogramming process. First of all, the choice of the somatic cell type influences the mutational load of the iPSCs. Older cells and cells that are frequently exposed to environmental factors such as UV light might have a higher mutational load than younger cells, such as cord blood cells. Since cord blood cells are easy to obtain and can be banked, they may provide an efficient source for clinical iPSCs. Furthermore, although using non-integrating delivery methods may reduce the risk of mutagenesis, several investigations have identified de novo mutations during reprogramming and culturing of iPSCs when reprogramming with both integrating and non-integrating methods [161, 162]. These genetic alterations result in variation amongst generated iPSCs. As discussed before, epigenetic modifications may also contribute to iPSC variation due to retained epigenetic memories of the starting cell type [163]. These variations may implicate differentiations of iPSCs towards the desired cell type. Low reprogramming efficiencies also remain an important issue for somatic cell reprogramming. Although RNA delivery may be a promising efficient non-integrating method, its reprogramming success rate is low and lentiviral delivery is therefore still amongst the most successful reprogramming method [164]. Addition to or substitution of the four Yamanaka factors have been shown to increase reprogramming efficiencies although not dramatically. Furthermore, several factors appear to be oncogenic and should therefore be chosen with careful consideration.

In summary, several aspects of the above-discussed reprogramming process should be taken into consideration when generating iPSCs (Fig. 1). iPSCs for clinical purposes will most likely have to be of higher quality and have to meet more stringent requirements than iPSCs for research purposes. Important choices will have to be made concerning the necessary reprogramming efficiencies and safety issues regarding the use of e.g., xenobiotic substances and integrated transgenes (e.g., oncogenes). The three parameters that influence the reprogramming process are the somatic cell type, the reprogramming factors and the delivery method. Epigenetic memory and the cell type’s susceptibility for infection or transfection may influence the choice of the somatic cell type. Furthermore, the endogenous expression levels of reprogramming factors in the chosen cell type and the possible side effects of the introduced factors (e.g., oncogenicity, off-target effects) influence the choice of the reprogramming factors. The state of the reprogramming factors (DNA, RNA, protein, small molecules) and the need for high reprogramming efficiencies or integration-free iPSCs will also affect the choice of the delivery method. Finally, improving culturing methods for maintenance of human iPSCs may increase overall reprogramming efficiencies and iPSC quality. Regardless the chosen methods, the generated iPSCs will obtain mutational load that still raises several safety issues which will have to be overcome before use of iPSC in clinical settings. Although other reprogramming methods such as SCNT may provide good alternatives, induced reprogramming remains to be the mostly commonly used and thoroughly characterized method. Taken together, it is recommended to carefully select the appropriate methods for the generation of iPSCs depending on their purposes.
